# The cognitive adaptability and resiliency employment screener (CARES): tool development and testing

**DOI:** 10.3389/fpsyt.2024.1430017

**Published:** 2024-07-10

**Authors:** Wilfredo Manuel R. Torralba, Marlyn Thomas Savio, Xieyining Huang, Priyanka Manchanda, Miriah Steiger, Timir Bharucha, María Martín López, Keanan J. Joyner, Rachel Lutz Guevara

**Affiliations:** ^1^ TaskUs Inc., New Braunfels, TX, United States; ^2^ Department of Psychology, University of California, Berkeley, Berkeley, CA, United States

**Keywords:** content moderator, factor analysis, psychometrics, recruitment, resilience

In the published article, there was an error in the **Abstract**, *Results*, for Phases 1 and 2. The number of items and CFI score were incorrectly reported. The sentence previously stated:

“In Phase 1, a set of 76 items were developed and tested via exploratory factor analysis, yielding three factors (i.e., Psychological Perseverance & Agility, Rumination & Emotional Lingering, and Expressiveness & Sociability) and also reducing the scale to 68 items. In Phase 2 through confirmatory factor analysis, the three-factor structure showed good fit (CFI = .92, RMSEA = .05) and demonstrated sufficient overall reliability.”

The corrected sentence appears below:

“In Phase 1, a set of 75 items were developed and tested via exploratory factor analysis, yielding three factors (i.e., Psychological Perseverance & Agility, Rumination & Emotional Lingering, and Expressiveness & Sociability) and also reducing the scale to 67 items. In Phase 2 through confirmatory factor analysis, the three-factor structure showed good fit (CFI = .93, RMSEA = .05) and demonstrated sufficient overall reliability.

In the published article, there was an error in **2. Phase 1**, Paragraph 8 for the number of items reported. This sentence previously stated:

“Next, by means of a live voting session, only items that reached consensus among the researchers were retained, resulting in a total of 76 items (Appendix 1).”

The corrected sentence appears below:

“Next, by means of a live voting session, only items that reached consensus among the researchers were retained, resulting in a total of 75 items (Appendix 1).”

In the published article, there was an error in **2.1.2. Measures**, Paragraph 1. The number of scale items was incorrectly reported. The sentence previously stated:

“The Cognitive Adaptability and Resiliency Employment Screener (CARES) is the 76-item employment screener developed by the authors to gauge the cognitive and psychological qualities essential to content moderation (Appendix 1).”

The corrected sentence appears below:

“The Cognitive Adaptability and Resiliency Employment Screener (CARES) was created from a pool of 75 items in an employment screener developed by the authors at the company to gauge the cognitive and psychological qualities essential to content moderation (Appendix 1).”

In the published article, there was an error in **2.1.2. Measures**, Paragraph 1. The number of items were incorrectly reported. The sentence previously stated:

“A total of 16 items focused on emotion regulation (e.g., “I prefer not to tell others what I am feeling,” “It is difficult for me to not get overwhelmed”), 10 items on cognitive factors (e.g., “I have a difficult time adjusting to last minute changes,” reverse; “I am able to set aside unwanted thoughts”), 8 items on grit (e.g., “Difficulties do not discourage me”), 10 items on optimism (e.g., “I maintain positivity even when others around me are not.”), 8 items on impulsivity (e.g., “I have a tendency to express my emotions immediately”), 10 items on neuroticism (e.g., “I do not let myself become ‘stuck’ on past events,” reverse), and 14 items on fear and worry response (e.g., “It is easy for me to let go of worrisome thoughts,” reverse).”

The corrected sentence appears below:

“A total of 24 items focused on emotion regulation (e.g., “I prefer not to tell others what I am feeling,”), 10 items on cognitive factor (e.g., “I have a difficult time adjusting to last minute changes”), 10 items on neuroticism/impulsiveness (e.g., “I do not let myself become ‘stuck’ on past events”), 10 items on optimism (e.g., “I maintain positivity even when others around me are not”), 7 items on grit (e.g., “Difficulties do not discourage me”), and 14 items on fear and worry response (e.g., “It is easy for me to let go of worrisome thoughts”).”

In the published article, there was an error in **2.1.3. Analysis Plan: EFA Model Specification**, Paragraph 1. The number of items was incorrectly reported. The sentence previously stated:

“In the Phase 1 data (*n* = 3,356), an EFA with the 76 potential CARES items was conducted using the ‘*psych*’ package in R (v2.3.3) (51), using maximum likelihood estimation with promax rotation.”

The corrected sentence appears below:

“In the Phase 1 data (*n* = 3,356), an EFA with the 75 potential CARES items was conducted using the ‘*psych*’ package in R (v2.3.3) (51), using maximum likelihood estimation with promax rotation.”

In the published article, there was an error in **2.2. Results and discussion**, Paragraph 1. Rerunning the analyses led to a reduction in cross-loadings and minor variations in the factor composition in the 12-factor structure. The sentence previously stated:

“The three-factor model produced a cleaner version of the test with fewer items with cross loadings (i.e., 10 items in the three-factor model with notable cross loadings vs. 18 items in the 12-factor model). Additionally, one of those factors in the 12-factor structure showed only 1 indicator.”

The corrected sentence appears below:

“Unlike the 12-factor model, the three-factor model produced a cleaner version of the test with no items with cross loadings. Additionally, two of those factors in the 12-factor structure did not evidence any indicators without cross-loadings, leaving no unique items for those factors.”

In the published article, there was an error in **2.2. Results and discussion**, Paragraph 1. Rerunning the analyses with the correct number of items changed the numerical values of variance and reliability estimates. The sentence previously stated:

“The final three-factor model retained 68 items, explaining approximately 39% of the total variance of all items. The three factors also functioned well as sum scores as indicated by high internal consistency reliability estimates (first factor: α = .96, ω = .97; second factor: α = .93, ω = .94; third factor: α = .76, ω = .87).”

The corrected sentence appears below:

“The final three-factor model retained 67 items, explaining approximately 38% of the total variance of all items. The three factors also functioned well as sum scores as indicated by high internal consistency reliability estimates (first factor: α = .96, ω = .97; second factor: α = .94, ω = .94; third factor: α = .77, ω = .87).”

In the published article, there was an error in **2.2. Results and discussion**, Paragraph 2. The means and standard deviations reported were not relevant to the revised analyses. The sentence previously stated:

“After evaluating the content of items on each of the factors, we named Factor 1 as Psychological Perseverance and Agility (PPA) (*Mean* = 135.51, *SD* = 16.39), Factor 2 as Rumination and Emotion Lingering (REL) (*Mean* = 68.16, *SD* = 19.67), and Factor 3 as Expressiveness and Sociability (ESc) (*Mean* = 21.55, *SD* = 4.55). PPA includes 31 questions, with REL including 30 questions and ESc including seven questions (see **Appendix 4** for details).”

The corrected sentence appears below:

“After evaluating the content of items on each of the factors, we named Factor 1 as Psychological Perseverance and Agility (PPA), Factor 2 as Rumination and Emotion Lingering (REL), and Factor 3 as Expressiveness and Sociability (ESc). PPA includes 30 questions, with REL including 30 questions and ESc including seven questions (see **Appendix 4** for details).”

**Figure 1:**
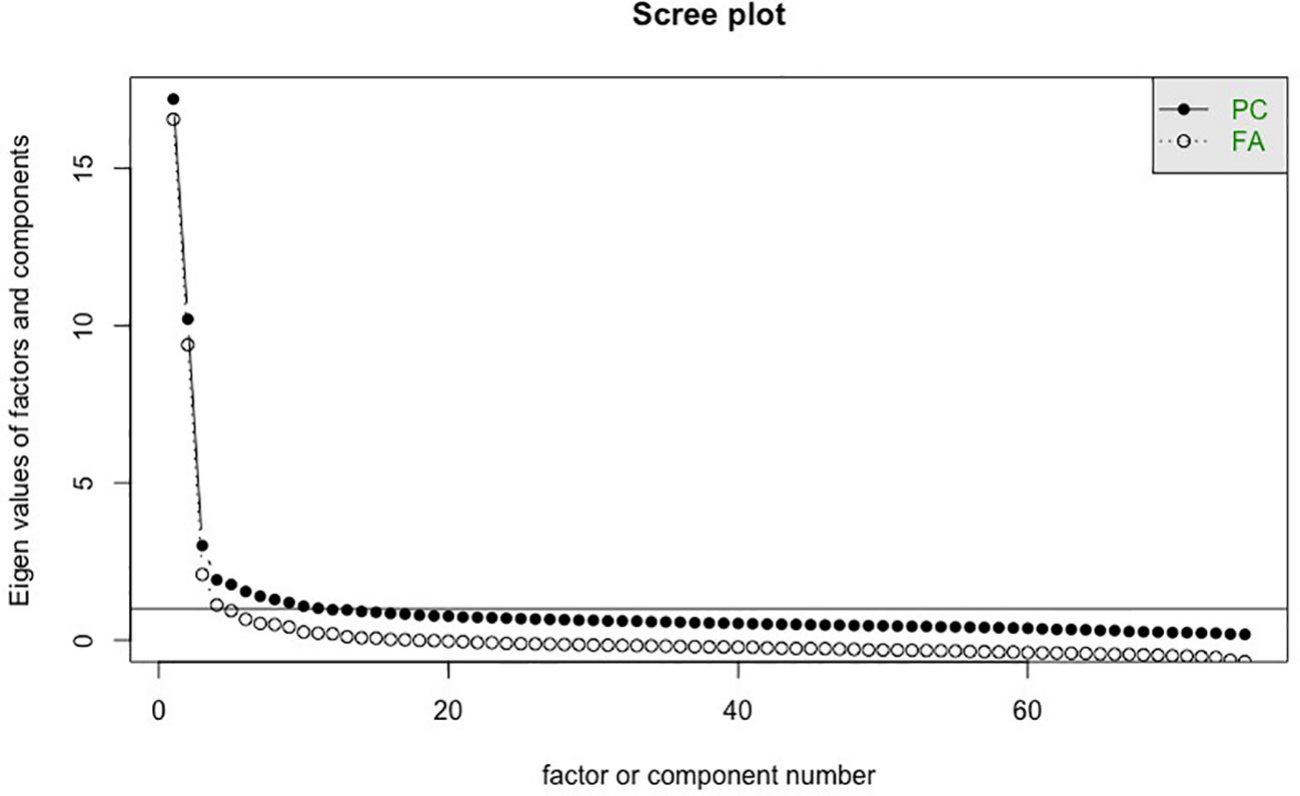
Scree plot for retaining factors (Exploratory Factor Analysis). PC, principal component; FA, factor analysis.

In the published article, there was an error in **Figure 1**. After publication, we noticed that one item, Grit 8, was a repetition of Cognitive Factor 1 (“I am able to prioritize and focus on important tasks.”). We reran Exploratory Factor Analysis without Grit 8 which led to a revision in the scree plot.

In the published article, there was an error in **3.1.2. Analysis Plan: CFA Model Specification**, Paragraph 1. The number of items was incorrectly reported. The sentence previously stated:

“A CFA with the 68 retained CARES items was conducted using the ‘*psych*’ package in R (v2.3.3) (51).”

The corrected sentence appears below:

“A CFA with the 67 retained CARES items was conducted using the ‘*psych*’ package in R (v2.3.3) (51).”

**Figure 2:**
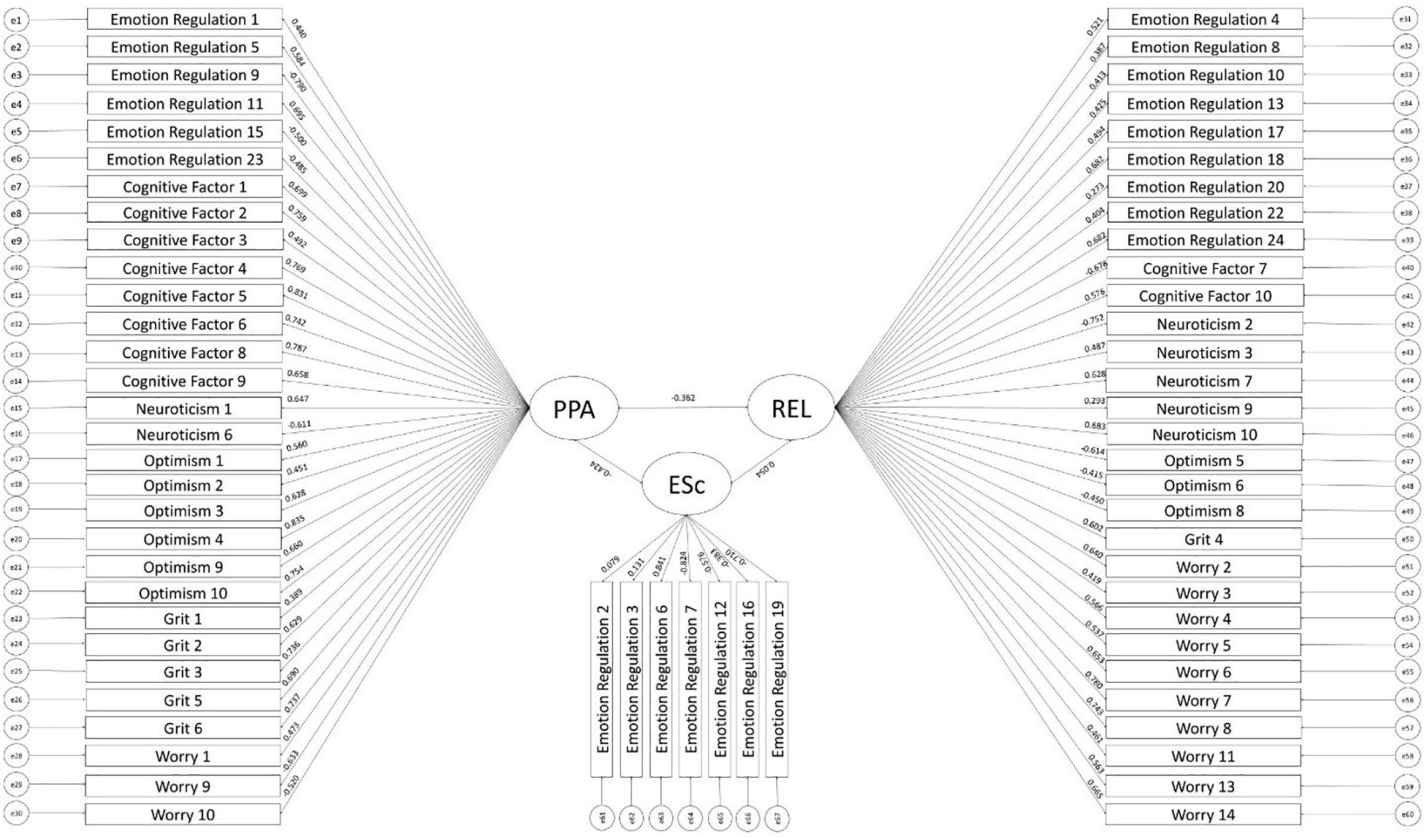
Three-factor model (confirmatory factor analysis). PPA, Psychological Perseverance & Agility; REL, Rumination and Emotional Lingering; ESc, Expressiveness and Sociability.

In the published article, there was an error in **3.2. Results and discussion**, Paragraph 1. On rerunning analyses, numerical values changed across the paragraph. The paragraph previously stated:

“Using diagonally weighted least squares (DWLS) estimation due to the ordinal nature of the items, the hypothesized three-factor model fit the data well, *χ* ^2^(2207) = 8,516.43, *p* <.001, CFI = 0.915, TLI = 0.912, RMSEA = .05, SRMR = .07 (**Figure 2**). Factor loadings were generally moderate-to-high and even across factors (PPA: mean λ = .64; REL: mean λ = .54; ESc: mean λ = .51). Replicating Study 1, the three factors also functioned well as sum scores as indicated by high internal consistency reliability estimates in Study 2 as well (PPA: α = .96, ω = .96; REL: α = .93, ω = .94; ESc: α = .75, ω = .86). Additionally, the average variance extracted (AVE) for the factors reflect varying levels of variance the latent construct accounts for in the manifest indicators. Specifically, PPA exhibited the highest AVE of.41, while REL and ESc demonstrated more moderate AVE of .29 and .26 respectively.”

The corrected paragraph appears below:

“Using diagonally weighted least squares (DWLS) estimation due to the ordinal nature of the items, the hypothesized three-factor model fit the data well, *χ* ^2^(2141) = 7360.53, *p* <.001, CFI = .928, TLI = .926, RMSEA = .05, SRMR = .07 (**Figure 2**). Factor loadings were generally moderate-to-high and even across factors (PPA: mean |λ| = .64; REL: mean |λ| = .55; ESc: mean |λ| = .51). Replicating Study 1, the three factors also functioned well as sum scores as indicated by high internal consistency reliability estimates in Study 2 as well (PPA: α = .96, ω = .96; REL: α = .93, ω = .94; ESc: α = .76, ω = .87). Additionally, the average variance extracted (AVE) for the factors reflect varying levels of variance the latent construct accounts for in the manifest indicators. Specifically, PPA exhibited the highest AVE of .39, while REL and ESc demonstrated more moderate AVE of .30 and .33 respectively.”

In the published article, there was an error in **Figure 2**. One item, Grit 8, was a repetition of Cognitive Factor 1 (“I am able to prioritize and focus on important tasks.”). We reran Confirmatory Factor Analysis without Grit 8, which led to changes in the numerical values at second decimal place and the movement of two items between factors (Emotion Regulation 8 moved from ESc to REL, and Emotion Regulation 16 moved from REL to ESc).

In the published article, there were errors in **4.2. Results and discussion**, Paragraphs 1 and 2. On rerunning analyses, numerical values changed across the paragraphs. The two paragraphs previously stated:

“As seen in **Table 1**, PPA showed good convergence with resilience (r(525) = .62) and cognitive control and flexibility (r(525) = .75). With respect to REL, it showed negative correlations with resilience (r(525) = −.52), cognitive control and flexibility (r(525) = −.70), and worry (r(525) = .66). REL showed a positive correlation with impulse strength (r(525) = .50), a subscale of BEQ. In terms of Esc, the associations were overall weaker compared with PPA and REL. The strongest observed correlation was between Esc and positive expressivity (r(525) = −0.40), a subscale of BEQ. Nonetheless, Esc demonstrated consistent (albeit smaller) correlations across different measures.

**Table 1:** Convergent and divergent validity.

Variables	PPA	REL	ESc
CD-RISC 10	0.62	−0.52	−0.26
CCFQ	0.75	−0.71	−0.36
Cognitive Control Over Emotions	0.62	−0.75	−0.37
Appraisal Coping Flexibility	0.73	−0.51	−0.27
LOT-R	0.37	−0.41	−0.29
DWQ	−0.44	0.66	0.30
BEQ	−0.10	0.40	−0.08
Negative Expressivity	−0.34	0.44	0.07
Positive Expressivity	0.25	−0.01	−0.31
Impulse Strength	−0.16	0.48	−0.06
Cognitive Reappraisal	0.51	−0.35	−0.18
Expressive Suppression	0.08	0.06	0.28

PPA, Psychological Perseverance & Agility; REL, Rumination and Emotional Lingering; Esc, Expressiveness and Sociability; CD-RISC 10, Connor Davidson Resilience Scale 10; CCFQ, Cognitive Control and Flexibility Questionnaire; LOT-R, Life Orientation Test Revised; DWQ, Dunn Worry Questionnaire; BEQ, Berkeley Expressivity Questionnaire; ERQ, Emotion Regulation Questionnaire.

In terms of divergent validity, PPA showed divergent validity with BEQ overall as well as its subscale Expressive Suppression (r(525) = .08; **Table 1**). Similarly, REL was uncorrelated with two BEQ subscales, namely, Positive Expressivity (r(525) = .03) and Expressive Suppression (r(525) = .03). Lastly, Esc was uncorrelated with two other BEQ subscales: Negative Expressivity (r(525) = −.02) and Impulse Strength (r(525) = −.03).”

The corrected paragraphs appear below:

“As seen in **Table 1**, PPA showed good convergence with resilience (r(524) = .62) and cognitive control and flexibility (r(524) = .75). With respect to REL, it showed negative correlations with resilience (r(524) = −.52), cognitive control and flexibility (r(524) = −.71), and a positive correlation with worry (r(524) = .66) and impulse strength (r(524) = .48), a subscale of the BEQ. In terms of ESc, the associations were overall weaker compared with PPA and REL. The strongest observed correlation was between ESc and cognitive control and flexibility (r(524) = −0.37). Nonetheless, ESc demonstrated consistent (albeit smaller) correlations across different measures.

In terms of divergent validity, PPA showed divergent validity with BEQ overall as well as its subscale Expressive Suppression (r(524) = −.10; **Table 1**). Similarly, REL was uncorrelated with two BEQ subscales, namely, Positive Expressivity (r(524) = −.01) and Expressive Suppression (r(524) = .06). Lastly, ESc was uncorrelated with two other BEQ subscales: Negative Expressivity (r(524) = .07) and Impulse Strength (r(524) = .06).”

In the published article, there was an error in **Table 1**. In the original article, one item, Grit 8, was a repetition of Cognitive Factor 1 (“I am able to prioritize and focus on important tasks.”) under PPA. We reran convergent and divergent validity analyses without Grit 8 which resulted in different values at second decimal place.

In the published article, there was an error in **5. General discussion**, Paragraph 1. The number of items was incorrectly reported. The sentence previously stated:

“The resulting 3-factor scale with 68 items demonstrated adequate reliability and validity when tested on large samples from the Philippines.”

The corrected sentence appears below:

“The resulting 3-factor scale with 67 items demonstrated adequate reliability and validity when tested on large samples from the Philippines.”

In the published article, there was an error in the Supplementary material, *Appendix 1*. The original title was “CARES for EFA (76 items)”. The correct title is “CARES for EFA (75 items)”.

In the published article, there was an error in the Supplementary material, *Appendix 1*. The ‘Neuroticism/Impulsiveness’ and ‘Optimism’ sections have been moved up before ‘Grit’ section to reflect the correct order as in the CARES form used for data collection.

In the published article, there was an error in the Supplementary material, *Appendix* 1, Paragraph 1. The number of items for grit was incorrectly reported as 8. The correct number of items is 7.

In the published article, there was an error in the Supplementary material, *Appendix 1*, ‘Emotion Regulation’. Items 4 (“I am non-confrontational and tend to avoid arguments.”), 14 (“I tend to worry more about others’ needs versus my own.”), 20 (“It is difficult for me to not get excited.”), and 21 (“I take on the emotions of others I encounter or see.”) were based on former versions which were not used for data collection. Appendix 1 has been updated with the correct phrasing which reflects the actual CARES form used for data collection.

In the published article, there was an error in the Supplementary material, *Appendix* 1, ‘Cognitive Factor’. Items 3 (“After a problem is solved, I don’t think about it afterwards.”), 4 (“I can attend to multiple tasks without being distracted.”) and 5 (“I am able to set aside unwanted thoughts.”) were based on former versions which were not used for data collection. Appendix 1 has been updated with the correct phrasing which reflects the actual CARES form used for data collection.

In the published article, there was an error in the Supplementary material, *Appendix* 1, ‘Neuroticism/Impulsiveness’. Items 3 (“I do not like working on tasks that I do not find rewarding.”) and 4 (“I tend to avoid situations that make me feel anxious.”) were based on former versions which were not used for data collection. Appendix 1 has been updated with the correct phrasing which reflects the actual CARES form used for data collection.

In the published article, there were errors in the Supplementary material, *Appendix* 1, ‘Grit’. Item 8, “I am able to prioritize and focus on important tasks.”, is a repetition of item 1 under Cognitive Factor, and has now been omitted. Furthermore, item 4 (“I never give up even when presented with multiple challenges.”) was based on former versions which were not used for data collection. Appendix 1 has been updated with the correct phrasing which reflects the actual CARES form used for data collection.

In the published article, there was an error in the Supplementary material, *Appendix* 1, ‘Fear and Worry Response’. Item 11 (“While finishing a task, I begin to worry in anticipation.”) was based on former versions which were not used for data collection. Appendix 1 has been updated with the correct phrasing which reflects the actual CARES form used for data collection.

In the published article, there was an error in the Supplementary material, *Appendix 2*. The row containing “grit_8” has been removed as this item is a repetition of “ccfq_1”.

In the published article, there was an error in the Supplementary material, *Appendix 3*. The row containing “grit_8” has been removed as this is a repeated item.

In the published article, there was an error in the Supplementary material, *Appendix 4*. The original title was “CARES (68 items retained)”. The correct title is “CARES (67 items retained)”.

In the published article, there was an error in the Supplementary material, *Appendix 4*, ‘Factor 1: Psychological Perseverance and Agility (PPA)’. Item 28, “I am able to prioritize and focus on important tasks.”, is a repetition of item 7 in the same factor, and has now been omitted.

In the published article, there was an error in the Supplementary material, *Appendix* 4, ‘Factor 1: Psychological Perseverance and Agility (PPA)’. Items 9 (“After a problem is solved, I don’t think about it afterwards.”), 10 (“I can attend to multiple tasks without being distracted.”) and 11 (“I am able to set aside unwanted thoughts.”) were based on former versions which were not used for data collection. Appendix 4 has been updated with the correct phrasing which reflects the actual CARES form used for data collection.

In the published article, there was an error in the Supplementary material, *Appendix* 4, ‘Factor 2: Rumination and Emotion Lingering (REL)’. Items 1 (“I am non confrontational and tend to avoid arguments”), 7 (“It is difficult for me to not get excited.”), 13 (“I do not like working on tasks that I do not find rewarding.”), 20 (“I never give up even when presented with multiple challenges.”) and 28 (“While finishing a task, I begin to worry in anticipation.”) were based on former versions which were not used for data collection. Appendix 4 has been updated with the correct phrasing which reflects the actual CARES form used for data collection.

In the published article, there was an error in the Supplementary material, *Appendix 5*. On rerunning analyses, there was a change in the second-place decimals for ‘Factor 2 × Factor 1’ (0.296), ‘Factor 3 × Factor 1’ (0.305) and ‘Factor 3 × Factor 2’ (0.283). Appendix 5 has been updated.

The authors apologize for these errors, and they state that this does not change the scientific conclusions of the article in any way. The original article has been updated.

